# A systematic review with meta-analysis of gastroesophageal reflux disease and exacerbations of chronic obstructive pulmonary disease

**DOI:** 10.1186/s12890-019-1027-z

**Published:** 2020-01-08

**Authors:** Chunrong Huang, Yahui Liu, Guochao Shi

**Affiliations:** 1 Department of Pulmonary and Critical Care Medicine, Ruijin Hospital, Shanghai Jiao Tong University School of Medicine, 197, Rui Jin Er Road, Shanghai, 200025 People’s Republic of China; 2 Institute of Respiratory Diseases, Shanghai Jiao Tong University School of Medicine, 197, Rui Jin Er Road, Shanghai, 200025 People’s Republic of China

**Keywords:** Chronic obstructive pulmonary disease, Gastroesophageal reflux disease, Meta-analysis

## Abstract

**Background:**

Gastroesophageal reflux disease (GERD) was suggested to be associated with exacerbations of chronic obstructive pulmonary disease (COPD) in recent years. The aim of this study was to examine the association between GERD and COPD exacerbation through a meta-analysis.

**Methods:**

Databases including EMBASE, MEDLINE, and the Cochrane Central Register of Controlled Trials were searched with a systematic searching strategy for original articles**,** published until Jan 2019, without language restriction.

**Results:**

A total of 13,245 patients from 10 observational articles were included in the meta-analysis. The meta-analysis indicated that GERD is associated with increased risk of COPD exacerbation (OR: 5.37; 95% CI 2.71–10.64). Patients with COPD and GERD had increased number of exacerbation (WMD: 0.48; 95% CI: 0.31 to 0.65).

**Conclusions:**

The meta-analysis showed that there was a significant correlation between GERD and COPD exacerbation.

## Background

Chronic obstructive pulmonary disease (COPD) is a prevalent pulmonary disorder characterized by persistent airway inflammation and reversible airflow restriction, with at least 170 million people world-wide estimated to be affected in population studies [1]. COPD is estimated to rank the third most common leading cause of death worldwide by 2020 [2, 3]. The substantial mortality is associated with severe acute exacerbation of COPD (AECOPD) with worsening airway function and respiratory symptoms and requiring emergency department attendance or hospitalization. Thus, the average two episodes of acute exacerbation annually suffered by patients with COPD were undoubtedly responsible for increasing mortality and the high burden of public health care [4, 5].

GERD is one of the most common gastrointestinal ailments worldwide, defined as the abnormal reflux of gastric contents into the esophagus, leading to esophageal mucosal injury or reflux symptoms, featured with two most common symptoms heartburn and regurgitation [6]. In recent years, a number of studies suggested the impression of higher prevalence of GERD in patients with COPD [7–10]. In a descriptive, cross-sectional study, the data showed a higher proportion of GERD symptoms are present in COPD patients and it also showed that GERD is more common in severe COPD patients [7]. GERD is related to coughing, reduced lung function [11], higher bronchial reactivity [12]. In return, frequent coughing and use of β2-agonists are thought to exacerbate reflux [13]. Thus, a vicious circle is between GERD and symptoms and function of patients with COPD formed. Recently, GERD is suggested as a putative risk factor of exacerbation of patients with COPD, as evidenced by the results that the GERD is associated with a significantly higher exacerbation rate and increased risk of admission to an ICU and mechanical ventilation use among COPD patients [14]. Hence, we aimed to conduct a meta­analysis of available studies comparing the risk of exacerbation of COPD in individuals with and without GERD.

## Method

This meta-analysis was conducted in accordance with the guidelines of the Preferred Reporting Items for Systematic Reviews and Meta-analyses (PRISMA) statement.

### Search strategy and study selection

We searched studies in MEDLINE, EMBASE, and the Cochrane Central Register of Controlled Trials published up to Jan 24, 2019, with no language restrictions. The search strategy in MEDLINE was used as follows: (((“Gastroesophageal Reflux”[Mesh]) OR ((((((((((((((Gastric Acid Reflux) OR Acid Reflux, Gastric) OR Reflux, Gastric Acid) OR Gastric Acid Reflux Disease) OR Gastro-Esophageal Reflux) OR Gastro Esophageal Reflux) OR Reflux, Gastro-Esophageal) OR Gastroesophageal Reflux Disease) OR GERD) OR Reflux, Gastroesophageal) OR Esophageal Reflux) OR Gastro-oesophageal Reflux) OR Gastro oesophageal Reflux) OR Reflux, Gastro-oesophageal))) AND (((((((((((COPD) OR Chronic Obstructive Pulmonary Disease) OR COAD) OR Chronic Obstructive Airway Disease) OR Chronic Obstructive Lung Disease) OR Airflow Obstruction, Chronic) OR Airflow Obstructions, Chronic) OR Chronic Airflow Obstructions) OR Chronic Airflow Obstruction)) OR “Pulmonary Disease, Chronic Obstructive”[Mesh]).

Two reviewers independently screened the titles and abstracts of the identified studies, and further searched the full texts of potentially relevant publications. Discrepancies were resolved by consensus.

### Selection criteria

Included articles must fulfill the following criteria: 1. GERD diagnosis could be made clinically with either of the followings: a. Self-reports; b. Questionnaire: “Has a doctor ever told you that you had gastro-oesophageal reflux or heartbur?”; c. The frequency of scale for the symptoms of GERD (FSSG) questionnaire composed of 12 questions (Additional file 1: Table S1): never = 0, occasionally = 1, sometimes = 2, often = 3, and always = 4. Total score>8 points was defined as GERD; d. Mayo gastro-oesophageal reflux questionnaire (GORQ) or Mayo Clinic GERD questionnaire: It was composed of four sections assessed the occurrence of heartburn, acid regurgitation, chest pain, and dysphagia, respectively, during the prior year; a separate section assessed the influence of heartburn and acid regurgitation on lifestyle and health-care utilization; nine questions assessed various symptoms attributable to the upper digestive tract; five questions assessed respiratory complaints; additional questions assessed the visits to a physician, hospitalizations for any reason during the prior year, medication history, medical history, family history of the disease, smoking and drinking status, and so on. Those who had daily or weekly GERD symptoms were considered as GERD positive. Apart from clinical diagnosis, GERD could also be diagnosed with any objective diagnostic method such as pH-metry (DeMeester score > 14.7). 2. COPD was defined as follows: post-bronchodilator ratio of forced expiratory volume in 1 s/forced vital capacity (FEV1/FVC) of < 70% in a patient with a smoking history of>10 pack-years. In some cases, patients with FEV1/FVC <88% pre after bronchodilator use and no response to bronchodilator (albuterol, 400 mcg) were also included. 3. Articles included patients with exacerbations of COPD. 4. Original cross-sectional, case-control or cohort studies provided sufficient information to calculate odds ratios (ORs) and 95% confidence intervals (CIs). Conference abstracts, case reports, editorials, clinical commentaries, and narrative reviews were disregarded.

### Data extraction

Two reviewers (Huang and Liu) independently extracted following information from all articles selected for inclusion in the meta-analysis: first author’s name, type and source of study design, age and gender of participants, smoking history, the sample size, criteria for GERD and COPD diagnosis, definition of COPD exacerbations, lung function, mean number of COPD exacerbations and hospitalization, rate of annual exacerbations.

### Assessment of quality and the risk of bias in the included studies

The Newcastle-Ottawa Scale (NOS) was used to evaluate the quality of included cohort studies and this includes eight assessment items for quality appraisal including ‘selection’, ‘comparability’ and ‘outcome’. According to the NOS score standard, cross-sectional studies could be classified as low-quality (scores of 0–4), moderate-quality (scores of 5–6) and high-quality (scores ≥7).

The methodological quality of the cross-sectional studies included was assessed using an 11-item checklist which was recommended by Agency for Healthcare Research and Quality (AHRQ). Article quality was assessed as follows: low quality = 0–3; moderate quality = 4–7; high quality = 8–11.

### Statistical analysis

Meta-analyses were performed using Review Manager 5.3 (Cochrane Collaboration, London, England). The program computed the weighted mean difference (WMD) and calculated 95% confidence interval (95% CI) for continuous variables under fixed-effects model, odds ratio (OR) and 95% CI were pooled for binary variables. Once significant heterogeneity existed, the random-effects model was performed to assess effect-size estimates.

Q statistic and I^2^ test were performed to estimate heterogeneity. A value of I2 of 0–25% represents insignificant heterogeneity, 26–50% represents low heterogeneity, 51–75% represents moderate heterogeneity, and > 75% represents high heterogeneity [15]. *P* value < 0.05 was considered as statistically significant heterogeneity. The risk of publication bias was evaluated by funnel plot.

Cochrane Q statistic and I^2^ test were both conducted to evaluate research heterogeneity among all the individual studies. Significant heterogeneity occurred if *p* < 0.05 or I^2^ > 50%, then, a random effects model would be chosen to pool the effect size. Otherwise, a fixed effect model would be used.

The sensitivity analysis was performed to assess the impact of study quality issues on the overall effect estimate and the effect size of these studies when neglecting heterogeneity and publication status.

## Results

### Literature search, characteristics and quality assessment

**Fig. 1:**
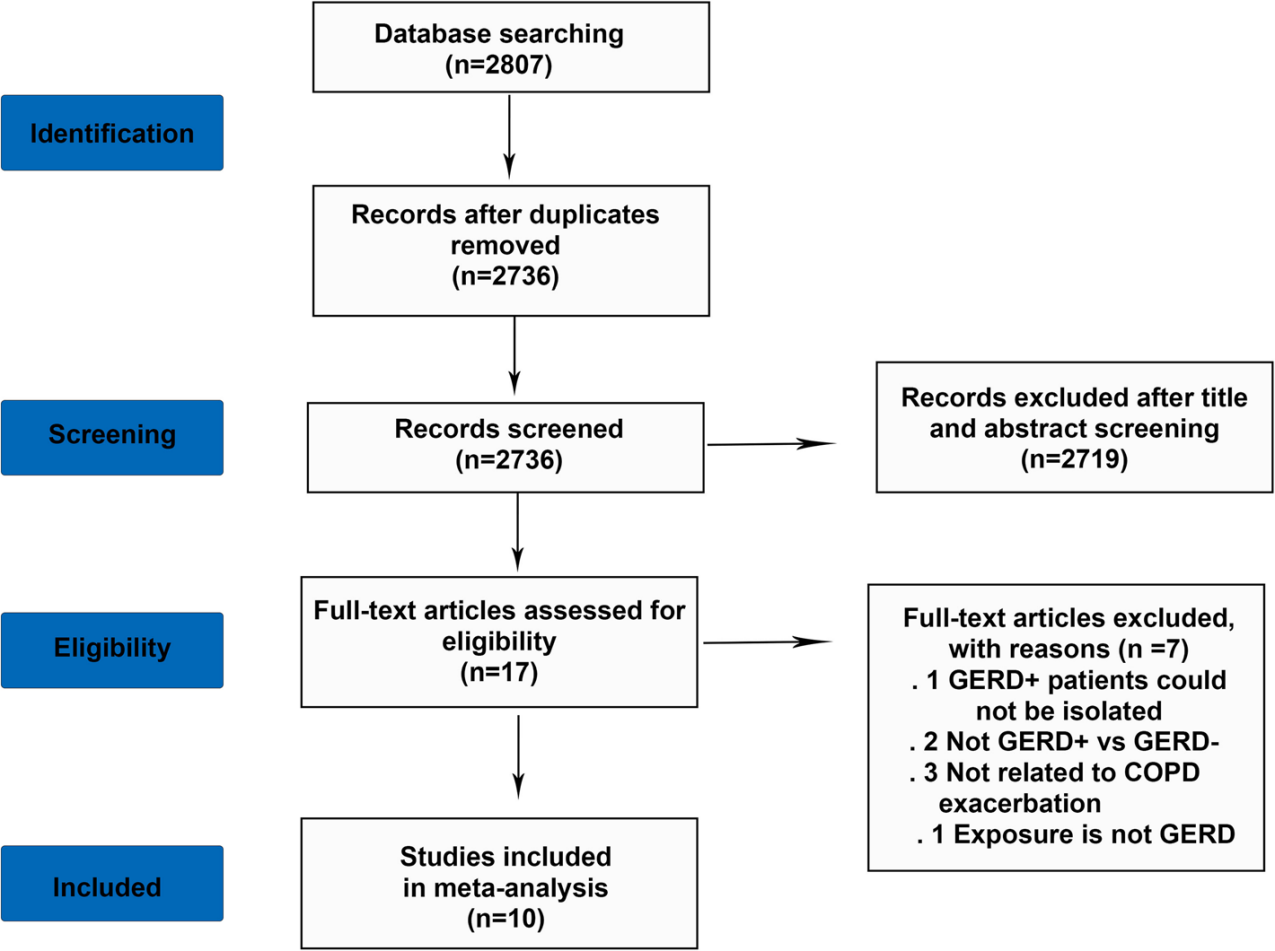
Flow chart of the selection of studies in this meta-analysis

In our study, a total of 2807 articles were identified from aforementioned databases, there were 2376 articles remaining after duplications were removed. After abstract and title screening, 2719 records were excluded because they were reviews, conference documents, editorials. Of the 17 articles selected for detailed evaluation, 7 were excluded because GERD+ patients could not be isolated (1), the comparison is not GERD+ vs GERD- in COPD patients (2), they didn’t involve the COPD exacerbation (3), exposure is not GERD (1). Ultimately, 10 articles were included in the meta-analysis [8, 9, 16–23], selection process was shown in Fig. 1.

Table 1 summarized the characteristics of the 10 included studies. Of these, three studies were conducted in America, one in Europe and six in Asia. Records from Japan [8, 9, 20, 21], Taiwan [17], Iran [22] were defined as Asian studies, while those from Europe [18] and America [16, 19, 23] were defined as Western studies. Sample size ranged from 48 to 5912 patients, and the meta-analysis consisted with a total sample size of13245 including 9 cohort studies and 2 cross-sectional studies. Only one article used 24-h pH monitoring to diagnose GERD [16].

**Table 1:** Primary Studies Included in the Meta-analysis

Study	Country	Design	Case Subjects	Control Subjects	Method of GERD Diagnosis	Method of COPD Diagnosis	Criteria of COPD exacerbation	Follow up
Bigatao et al. (2018) [16]	United States	cohort study	21 COPD patients with GERD	27 COPD patients without GERD	pH-metry: DeMeester score > 14.7	FEV1/FVC <88% pre after bronchodilator use and no response to bronchodilator (albuterol, 400 mcg)	occurrence of increase in respiratory symptoms [(dyspnea, cough, and sputum (purulent or not)] that required the use of antibiotics and/or oral corticosteroids	12 months
Lin et al. (2015) [17]	Taiwan	cohort study	1976 COPD patients with GERD	3936 COPD patients without GERD	NOT stated	The diagnosis of COPD was identified based on the International Classification of Diseases, 9th Revision, Clinical Modification codes (ICD-9-CM codes 491, 492, 496)	COPD-related ED admission or hospitalisation during which the subject received bronchodilators or steroids during the one-year follow-up.	12 months
Benson et al. (2015) [18]	United Kingdom	cohort study	547 COPD patients with GERD	1558 COPD patients without GERD	Questionnaire	smoking history ≥10 pack years, a post-bronchodilator Forced Expiratory Volume in 1 s (FEV1) < 80% of predicted value, and FEV1/FVC ≤ 0.7	Patients treated with antibiotics and/or systemic corticosteroids, or requiring hospitalisation were included	3 years
Martinez et al. (2014) [19]	United States	cross -sectional study	1307 COPD patients with GERD	3176 COPD patients without GERD	Self-report of physician-diagnosed GERD	met criteria for GOLD stage 1 or greater (fixed airflow obstruction with a post-bronchodilator FEV1/FVC ≤ 0.7), CT measurements of emphysema and airway abnormalities	ATS Chronic Respiratory Disease Questionnaire (ATS-DLD-78)	NOT stated
Shimizu et al. (2012) [20]	Japan	cohort study	40 COPD patients	40 control subjects	The frequency of scale for the symptoms of GERD (FSSG) questionnaire: total score>8 points	GOLD criteria	worsening that required an unscheduled visit to the local doctor, emergency department, or hospital, or else needed treatment with oral or intravenous corticosteroids at least one episode during the past two years	Not stated
Takada et al. (2011) [21]	Japan	cohort study	59COPD patients with GERD	162 COPD patients without GERD	FSSG questionnaire: total score>8 points	symptoms of chronic sputum or dyspnea on effort and FEV1/FVC < 70% after use of a bronchodilator	AECOPD was defined based on symptoms of Anthonisen type 1 or 2 and prescription of additional systemic corticosteroids or antibiotics	1 year
Terada et al. (2010) [9]	Japan	cohort study	67 patients with COPD	19 age-matched controls	FSSG questionnaire: total score>8 points	Not stated	the presence of an increase in any two major symptoms (dyspnea, sputum purulence, and sputum quantity) or an increase in one major and one minor symptom (wheeze, sore throat, cough, and nasal congestion/discharge) for at least two consecutive days	12 months
Rogha et al. (2010) [22]	Iran	cohort study	59 COPD patients with GERD	51 COPD patients without GERD	Mayo gastro-oesophageal reflux questionnaire (GORQ)	FEV1/FVC < 0.7,age ≥ 40 years, and a ≥ 20 pack-year history of smoking or history of exposure to occupational dusts or chemicals for ≥10 years	increase in cough frequency and severity, increase in dyspnea, or change in the amount and/or character of sputum	1 year
Terada et al. (2008) [8]	Japan	cohort study	82 patients with COPD	40 controls	FSSG questionnaire: total score>8 points	(GOLD) 2003	the occurrence of two or more of three major symptoms (ie, increase in dyspnoea, sputum purulence and increased sputum volume), or any one major symptom with any one minor symptom (ie, increase in nasal discharge, wheezing, sore throat, cough or fever) for at least 2 consecutive days	Over 6 months
Rascon-Aguilar et al. (2006) [23]	United States	cross -sectional survey	32 patients with COPD	54 COPD patients without GERD	Mayo Clinic GERD questionnaire	FEV1/FVC ratio ≤ 70% on pulmonary function tests (PFTs),age ≥ 40 years, and a ≥ 20 pack-year history of smoking	worsening dyspnea, increasing volume of sputum, or purulent sputum in conjunction with physician-initiated use of corticosteroids or antibiotics, hospitalization, or emergency department (ED) visit during the previous 12 months	

The quality scores of cohort studies ranged from 5 to 7 with an average score of 8.75 (Additional file 1: Table S2). Four studies were evaluated with a score of < 7, and others with a score of ≥7 (Additional file 1: Table S3). Two cross-sectional studies exhibited moderate quality. Thus, the majority of the studies included in the meta-analysis were assessed as moderate-high-quality studies.

### Main analysis of the association between GERD and exacerbation of COPD

**Fig. 2:**
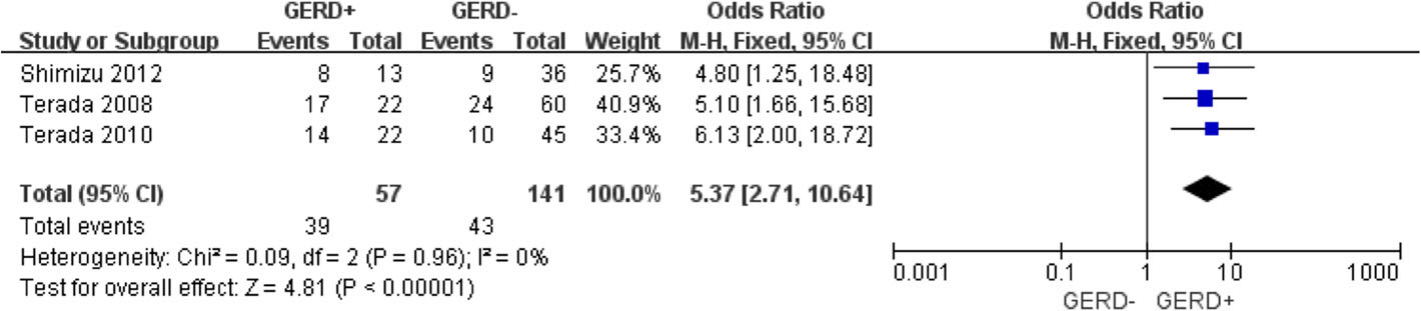
Forest plot of odds ratios for the frequency of COPD exacerbation in patients with GERD compared those without GERD

Meta-analytic pooling for COPD exacerbations showed that GERD significantly increased the risk of COPD exacerbation (OR: 5.37; 95% CI 2.71 to 10.64; *p* = 0.96, I^2^ = 0%; Fig. 2).

**Fig. 3:**
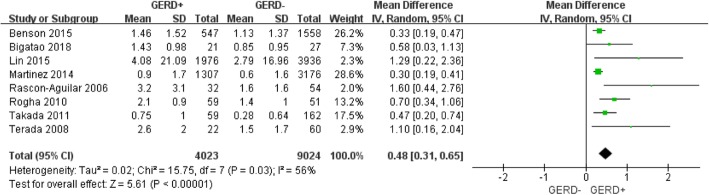
Forest plot of mean difference of frequency of exacerbation in COPD patients with and without GERD

**Fig. 4:**
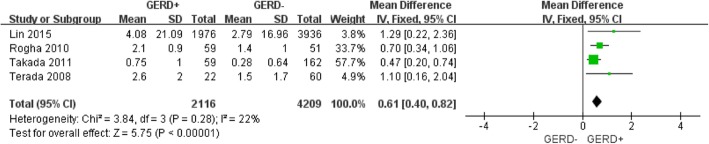
Forest plot of mean difference in exacerbations in COPD patients with and without bronchiectasis in Asian countries

Figure 3 showed that COPD patients with GERD exhibited more exacerbations (WMD: 0.48 times per year; 95% CI: 0.31 to 0.65; *P* = 0.03; I^2^ = 56%; Fig. 3) than COPD patients without GERD, however there was observable publication bias (Additional file 1: Figure S1) and statistical heterogeneity. Of note, the diagnostic methods for GERD varied across studies, but only one study relied on objective methods [16], 24-h pH monitoring, in this study, GERD patients exhibited increased the frequency of exacerbation (WMD: 0.58 times per year; 95% CI: 0.03 to 1.13; *P* = 0.03; data not shown). Figure 4 shows a pooled analysis of the relationship between GERD patients and the frequency of COPD exacerbation in Asian areas, COPD patients with GERD showed a higher frequency of exacerbations (WMD: 0.618 times per year; 95% CI: 0.40 to 0.82; *P* = 0.28; I^2^ = 22%; Fig. 4), the above statistical heterogeneity vanished (56% vs 22%) when the factor was considered. It suggested that the geographical difference might be a factor related to the heterogeneity.

### Sensitivity analysis

When using fixed effect model, as shown in Additional file 1: Figure S2, the increased frequency of exacerbation in COPD patients with GERD was 0.37 times per year (95% CI: 0.29 to 0.44; *P* = 0.03; I^2^ = 56%) compared to those without GERD, and this result was similar to that observed using random effects model, indicating the stable results. The coupled forest plots showed moderate heterogeneity (I^2^ = 56%), but the results didn’t dramatically influenced the pooled results when we omit one study at each turn, fluctuating from 47 to 61% (Table 2). The results showed that the corresponding pooled ORs were not materially altered, indicating that our results were statistically robust.

**Table 2:** Sensitivity Analyses

Omitted Study	Mean(95% CI)	Heterogeneity
Bigatao (2018) [16]	0.48(0.30–0.66)	*P* = 0.05,I^2^ = 53%
Lin (2015) [17]	0.45(0.29–0.61)	*P* = 0.02,I^2^ = 60%
Benson (2015) [18]	0.60(0.35–0.86)	*P* = 0.02,I^2^ = 61%
Martinez (2014) [19]	0.60(0.36–0.84)	*P* = 0.05,I^2^ = 53%
Takada (2011) [21]	0.50(0.30–0.71)	*P* = 0.02,I^2^ = 60%
Rogha (2010) [22]	0.44(0.27–0.60)	*P* = 0.06,I^2^ = 51%
Terada (2008) [8]	0.46(0.29–0.62)	*P* = 0.04,I^2^ = 55%
Rascon-Aguilar (2006) [23]	0.44(0.29–0.58)	*P* = 0.08,I^2^ = 47%

## Discussion

In general, this meta-analysis of observational studies suggested GERD increased the risk of exacerbation of patients with COPD (OR = 5.37; 95% CI: 2.71 to 10.64), and patients of COPD with GERD exhibited more exacerbations (WMD: 0.48 times per year; 95% CI: 0.31 to 0.65). The sensitivity analysis revealed that after any individual study was omitted or the fixed-effects model was converted to a random-effects model, I^2^ ranged from 47 to 61%, the results and conclusions still exist. Therefore, we have some confidence to believe the result of association between GERD and exacerbation of COPD in our meta-analysis.

This current study is not the first meta-analysis to investigate the relationship between GERD and the exacerbation of COPD, previous meta-analysis identified GERD as a risk factor for COPD exacerbations (RR = 7.57; 95% CI: 3.84 to 14.94), with an increased mean number of exacerbations per year (mean difference: 0.79; 95% CI: 0.22 to 1.36), both of which were higher than that of current study [24]. There were some differences between previous study conducted by Sakae et al. and ours. Firstly, relative risk (RR) was used as the effect estimate in previous study to evaluate the risk of exacerbation in patients with COPD and GERD, therefore, the studies included were different from that of current meta-analysis. Secondly, the previous study published in 2013 incorporated of 2769 patients, smaller sample size than ours, which may overestimate the role of GERD in exacerbation of COPD to some extent. Thirdly, our study excluded the research by Eryuksel et al. [25], who investigated the relationship between Laryngopharyngeal reflux (LPR) and COPD, because of the different pathophysiology and symptoms of LPR from that of GERD [26]. Therefore, we performed the meta-analysis to reevaluate the relationship between GERD and the exacerbation of COPD.

Acute exacerbation of COPD often defined as worsening major symptoms, requiring antibiotics and/or systemic corticosteroids, or requiring hospitalization or emergency department admission. An exacerbation can affect the normal course of disease and severely reduce patients’ quality of life, being responsible for the main cause of high mortality rate in COPD patients [27]. In recent years, a growing number of evidence suggests a positive association between GERD and exacerbation. Rascon-Aguilar and colleagues [23] conducted a questionnaire-based, cross-sectional survey and found that the rate of exacerbations of COPD was twice as high in patients with GER symptoms compared to those without GER symptoms. Another prospective cohort study reported higher prevalence of gastro-oesophageal reflux disease (GORD) symptoms in COPD patients, and the frequency of exacerbations was significantly associated with the Frequency Scale for the Symptoms of GORD (FSSG) score, multiple regression analysis further revealed that GORD symptoms were significantly associated with the occurrence of exacerbations (RR = 6.55, 95% CI: 1.86 to 23.11) [8]. In a large cross-sectional and longitudinal study of 4483 participants, Martinez et al. found that patients with COPD and GERD were more susceptible to worse quality of life (QOL), dyspnea, and experience exacerbations during two-year-follow-up time compared with COPD patients without GERD [19]. It’s important to note that these data suggested GERD as a risk factor of the exacerbation of COPD without considering whether the patient was prescribed for acid inhibitory treatments or not. Ingebrigtsen et al. [28] reported the positive association between GERD and COPD exacerbations among patients without using acid inhibitory treatment regularly during 5-year-follow-up (hazards ratio (HR): HR = 2.7 (1.3 to 5.4, *P* = 0.006)), however, it’s not true in individuals using acid inhibitory treatment regularly, in another words, they had not an increased risk of exacerbations, HR = 1.2 (0.6 to 2.7, *P* = 0.63). Herein, pharmaceutical treatment is an important confounding factor affecting the potential association, we could easily speculate that GERD treatment might decrease the frequency of episodes of exacerbation. Yet, this association remained contradictory when acid inhibitory medications were taken into consideration, because some studies reported a reduction of exacerbation in patients with GERD using acid inhibitory medications [29], while some others demonstrated that proton-pump inhibitor medications (PPIs) use could be associated with more frequent exacerbations among patients with GER symptoms [19]. These data implied a potential association between GERD and exacerbations based on considerations of pharmaceutical prescription. In our meta-analysis, we couldn’t perform the subgroup analysis in terms of medications of GERD because of inadequate data in all the included studies. Therefore, it’s still unclear how far acid inhibitory treatment could affect the exacerbation of COPD. More definitive clinical trials are warranted to address their association, and attempt to solve the pressing problem for novel interventions to reduce exacerbations of COPD.

Exacerbations play a pivotal role in prognosis and mortality of COPD patients. However, the pathology of GERD in COPD is complex and obscure, and the mechanism by which GER symptoms affect COPD exacerbation remains to be elucidated. Some researches summarized the possible pathological mechanisms assumed to account for acute exacerbation in COPD patients with GERD. The primary pathophysiological basis lies on the rationale that reflux of (duodeno-) gastric contents (acid or non-acid components) cause significant airway irritation and injury, increasing bronchial reactivity changes and leading to pulmonary symptoms such as a tracheal or bronchial cough reflex [30, 31]. Another to note is same vagus innervation shared by the oesophagus and tracheal bronchus [32], stimulation of the which could cause bronchoconstriction through esophago-bronchial reflex and heightened cough reflex [33, 34]. Moreover, one of the most important aggravating factors in relation to exacerbations of COPD is respiratory infection [35]. Pulmonary inflammatory reaction because of aspirations of non-infectious chemicals or bacteria in the process of reflux or its related swallowing impairment is also an unnegligible factor favoring the development of exacerbations significantly [23, 36]. However, it’s not sufficient to draw the causal effect of GERD in exacerbations of COPD when we consider this issue in return. Sleep apnea, a common ailment existed in COPD patients, causes GER and non-acidic reflux [37]. Increased gastro-esophageal pressure gradient, recurrent coughing, diaphragmatic flattening in COPD patients are proposed to exacerbate reflux [13, 38]. In addition, β2-adrenergic agonists, a significant class of medications that have the potential to reduce exacerbations and related hospitalizations and improve overall health outcomes of COPD with the function to relax bronchial smooth muscle and relieve symptoms of bronchoconstriction [39], were reported to reduce lower esophageal sphincter tone, which is a primary pathophysiologic mechanisms suggested to account for GER [13, 38, 40].

Taken together, the above-mentioned researches suggest that GERD is not only a precipitating factor in the development of COPD, but that it can worsen ongoing and impact the outcomes of COPD as well. In spite of the potential impact on COPD exacerbations, it is difficult to establish a causal relationship since the manifestations, drugs or comorbidities in COPD may induce GERD in return.

There are some limitations in the meta-analysis that warrant mentioning. Firstly, four retrospective cohort studies and two cross-sectional studies were available, the subjects were asked to report the aspects of exacerbations during the previous year, which was a possible cause of a recall bias. And no cohort study in prospective design to date has been conducted utilizing standardized diagnoses to explore the impact of GERD on the outcome of exacerbations in COPD. Secondly, the results may be biased by different measurement techniques to diagnose GERD (reflux questionnaire/24-h pH monitoring), and pH monitoring was performed in only one study, lack of standardized patient-reported questionnaires may underestimate or overestimate the presence of GERD and distort their associations. Meanwhile, the diagnosis of COPD varied between some studies, such as the duration of smoking history, the ratio of FEV1/FVC. Additionally, subgroup analysis revealed the possible source of heterogeneity from geographical difference, we couldn’t explore heterogeneity from other aspects such as whether the acid suppression treatments in patients with GERD and COPD could affect the exacerbations, because of inadequate data reported. Furthermore, there are hurdles to decipher the role of other contributing factors in the study, especially the prevalent respiratory infection in COPD exacerbation, which warranted further investigation.

Despite these limitations, all of the studies included consisting of 13,245 patients, were assessed as moderate to high quality. And most of the studies were in cohort design. These strengths granted us some confidence to speculate an association between GERD and exacerbation of COPD. However, our results should be interpreted with caution and need further researches in the light of several limitations.

## Conclusions

In summary, high GERD risk appears to be associated with higher odds for exacerbations of COPD. The recognition of the relationship may help physicians to better monitor and prevent GERD in patients with COPD exacerbations.

## Supplementary information

**Additional file 1: Table S1.** Questions of the frequency of scale for the symptoms of GERD (FSSG). **Table S2.** Newcastle-Ottawa Scale of the Cohort Studies Included in the Meta-analysis. **Table S3.** Quality assessment of the cross-sectional studies Included in the Meta-analysis by Agency for Healthcare Research and Quality (AHRQ) *. **Figure S1.** Funnel plot of meta-analysis. **Figure S2.** Forest plot of mean difference of frequency of exacerbation in COPD patients with and without GERD under fixed model.

## Data Availability

The authors declare that all data supporting the findings of this study are available within the following articles and its supplementary information files.

## Abbreviations

COPD: Chronic obstructive pulmonary disease
GERD: Gastroesophageal reflux disease
OR: Odds ratio
PRISMA: Preferred Reporting Items for Systematic Reviews and Meta-Analyses
WMD: Weighted mean difference
