# Supplementary material for: A systematic review with meta-analysis of gastroesophageal reflux disease and exacerbations of chronic obstructive pulmonary disease
Source: BMC Pulm Med. 2020 Jan 8;20:2. doi: 10.1186/s12890-019-1027-z (PMC6950869; doi:10.1186/s12890-019-1027-z)

**Table S1. Questions of the frequency of scale for the symptoms of GERD (FSSG)**

| Questions |
| --- |
| 1. Do you get heartburn? |
| 2. Does your stomach get bloated? |
| 3. Does your stomach ever feel heavy after meals? |
| 4. Do you sometimes subconsciously rub your chest with your hand? |
| 5. Do you ever feel sick after meals? |
| 6. Do you get heartburn after meals? |
| 7. Do you have an unusual (e.g. burning) sensation in your throat? |
| 8. Do you feel full while eating meals? |
| 9. Do some things get stuck when you swallow? |
| 10. Do you get bitter liquid (acid) coming up into your throat? |
| 11. Do you burp a lot? |
| 12. Do you get heartburn if you bend over? |

**Table S2. Newcastle-Ottawa Scale of the Cohort Studies Included in the Meta-analysis**

|  | **Selection** | | | | **Comparability** | **Outcome** | | |  |
| --- | --- | --- | --- | --- | --- | --- | --- | --- | --- |
| **Study** | **Representativeness of the exposed cohort** | **Selection of the non-exposed cohort** | **Ascertainment of exposure** | **Outcome of interest was not present at start of study** | **Comparability of cohorts on the basis of the design or analysis1** | **Assessment of outcome** | **Follow-up long enough for outcomes to occur2** | **Adequacy of follow up of cohorts3** | **Total Score** |
| Bigatao (2018) | * | * | * | * | ** | * | - | - | 7 |
| Lin (2015) | * | * | - | * | * | * | - | - | 5 |
| Benson (2015) | * | * | * | * | ** | * | - | - | 7 |
| Shimizu (2012) | * | * | * | * | - | * | - | - | 5 |
| Takada (2011) | * | * | * | * | * | * | - | - | 6 |
| Terada (2010) | * | * | * | * | ** | * | - | - | 7 |
| Rogha (2010) | * | * | * | * | ** | * | - | - | 7 |
| Terada (2008) | * | * | * | * | - | * | - | - | 5 |

1 A maximum of 2 points could be allotted in this category. Studies that included adjustment for age, sex (if applicable) and smoking status or intensity received one point, and studies that included some of the other important confounders such as body mass index, alcohol, dietary factors received an additional point.

2 A cohort study with a follow-up time >5 years was assigned one point.

3 A cohort study with a follow-up participation rate >85% was assigned one point.

**Table S3. Quality assessment of the cross-sectional studies Included in the Meta-analysis by Agency for Healthcare Research and Quality (AHRQ) ***

| Item | Rascon-Aguilar (2006) | Martinez (2014) |
| --- | --- | --- |
| 1. Define the source of information (survey, record review) | 1 | 1 |
| 2. List inclusion criteria for exposed and unexposed subjects (cases and controls) or refer to previous publications | 1 | 1 |
| 3. Indicate time period used for identifying patients | 1 | 1 |
| 4. Indicate whether or not subjects were consecutive if not population based | 1 | 1 |
| 5. Indicate if evaluators of subjective components of study were masked to other aspects of the status of the participants | 0 | 0 |
| 6. Describe any assessments undertaken for quality assurance purposes (e.g. test/retest of primary outcome measurements) | 0 | 0 |
| 7. Explain any patient exclusion from analysis | 0 | 0 |
| 8. Describe how confounding was assessed and/or controlled | 0 | 1 |
| 9. If applicable, explain how missing data were handled in the analysis | 0 | 0 |
| 10. Summarize patient response rates and completeness of data collection | 0 | 0 |
| 11. Clarify what follow-up, if any, was expected and the percentage of patients for which incomplete data or follow-up was obtain | 0 | 0 |
| Total scores | 4 | 5 |

* The item would be scored ‘0’ if it was answered ‘NO’ or ‘UNCLEAR’; if it was answered ‘YES’, then the item scored ‘1’.

**Figure S1. Funnel plot of meta-analysis**


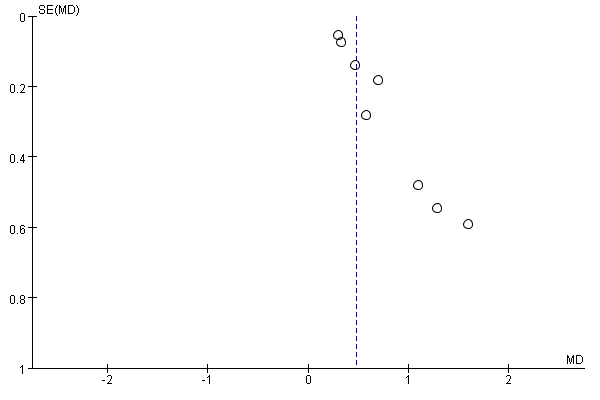


**Figure S2. Forest plot of mean difference of frequency of exacerbation in COPD patients with and without GERD under fixed model.**


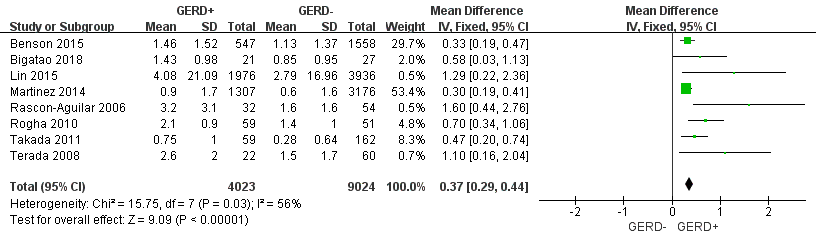

Supplement: Supplementary file 1 — Additional file 1: Table S1. Questions of the frequency of scale for the symptoms of GERD (FSSG). Table S2. Newcastle-Ottawa Scale of the Cohort Studies Included in the Meta-analysis. Table S3. Quality assessment of the cross-sectional studies Included in the Meta-analysis by Agency for Healthcare Research and Quality (AHRQ) *. Figure S1. Funnel plot of meta-analysis. Figure S2. Forest plot of mean difference of frequency of exacerbation in COPD patients with and without GERD under fixed model. [file 12890_2019_1027_MOESM1_ESM.docx]
